# Supplementary figures and images for: Developmental and aging changes in brain network switching dynamics revealed by EEG phase synchronization
Source: PLoS Comput Biol. 2026 Apr 16;22(4):e1013290. doi: 10.1371/journal.pcbi.1013290 (PMC13124065; doi:10.1371/journal.pcbi.1013290)

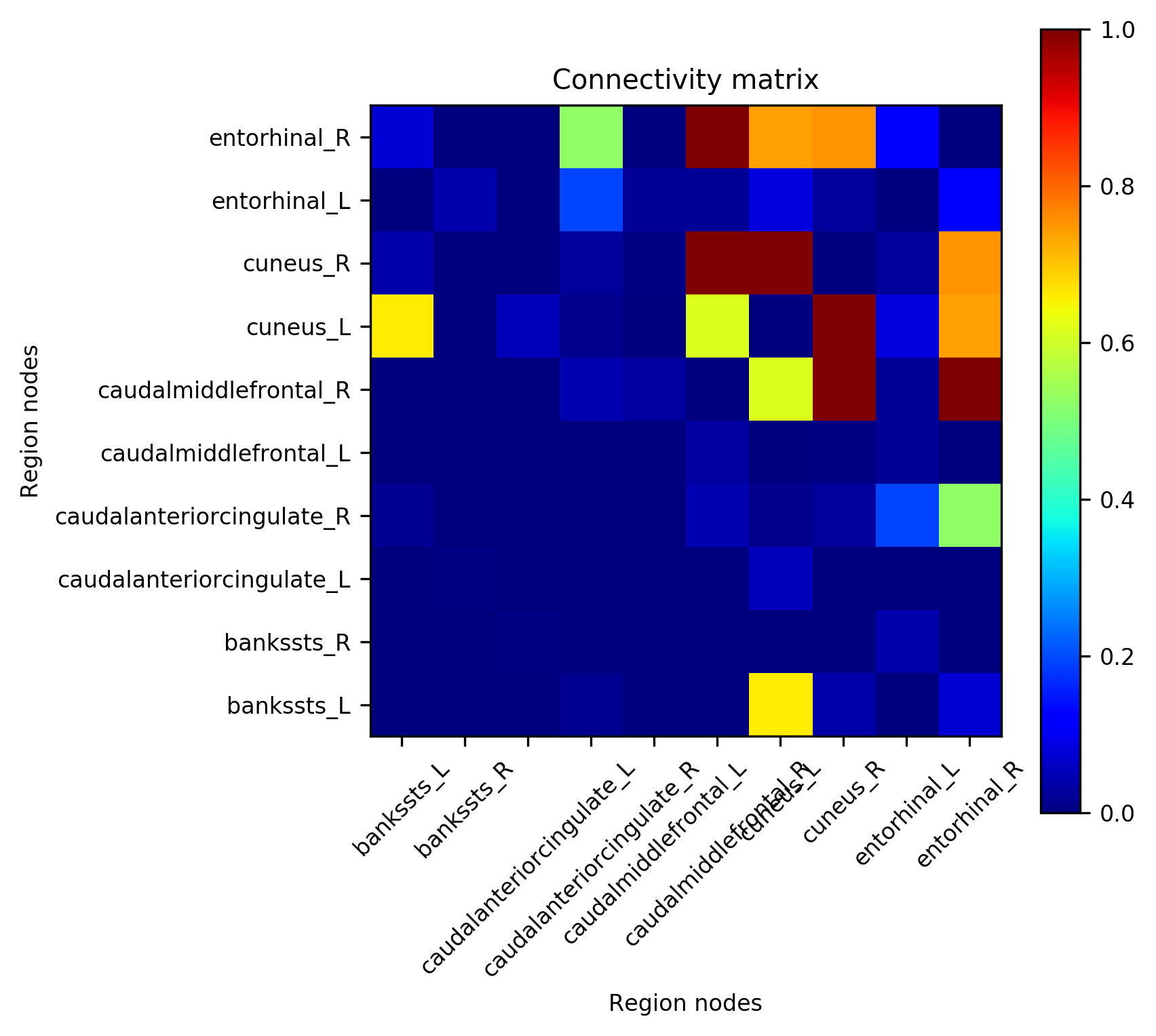


**S1 Fig. Connectivity used for simulations.**

Supplement: S1 Fig — (DOCX) [file pcbi.1013290.s001.docx]

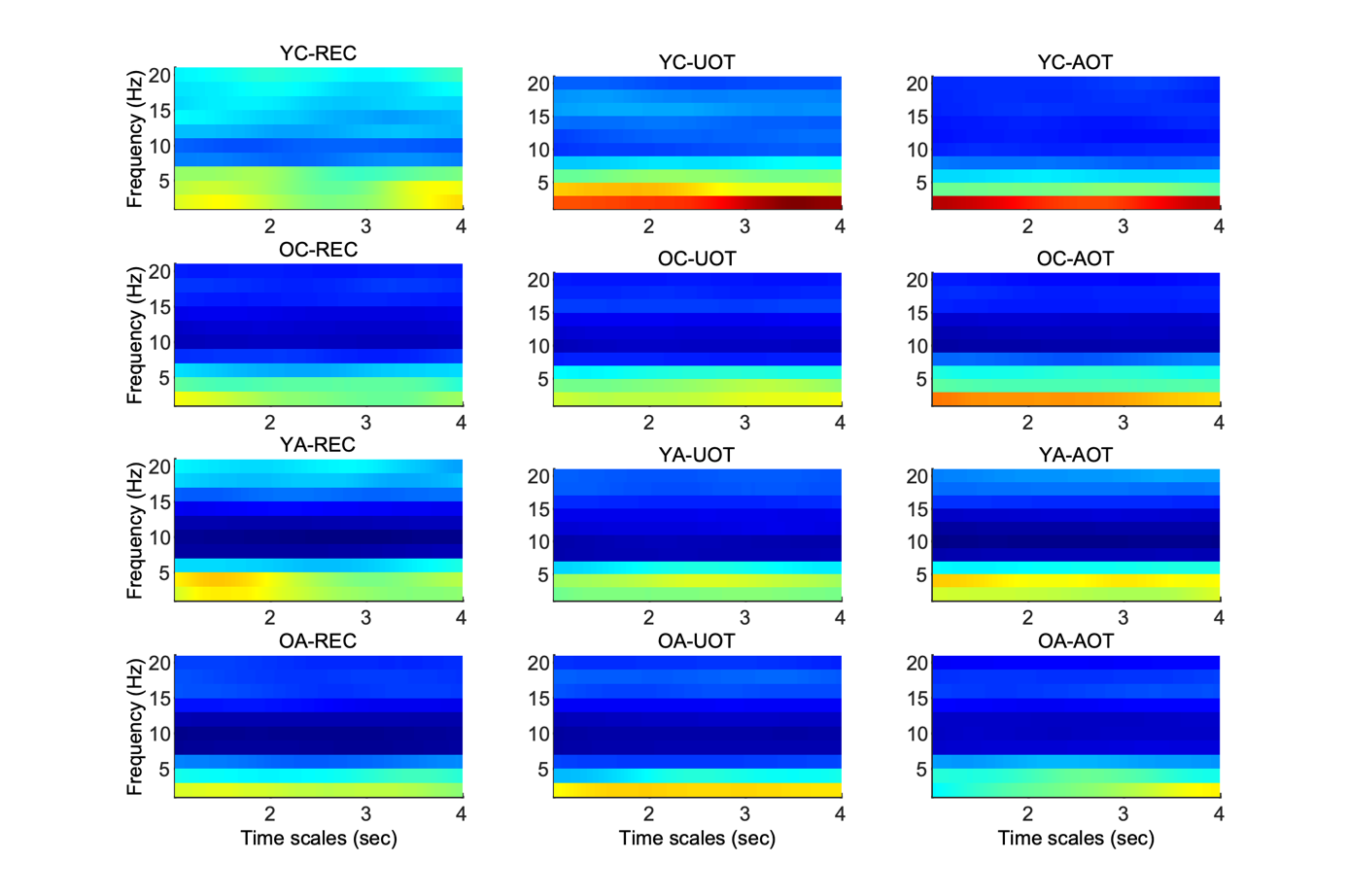


**S3 Fig. Group and condition t-score of *μJL*(τ,*f*).** Figure arrangement and conventions similar to **S2 Fig**

Supplement: S3 Fig — Figure arrangement and conventions similar to S2 Fig. (DOCX) [file pcbi.1013290.s003.docx]

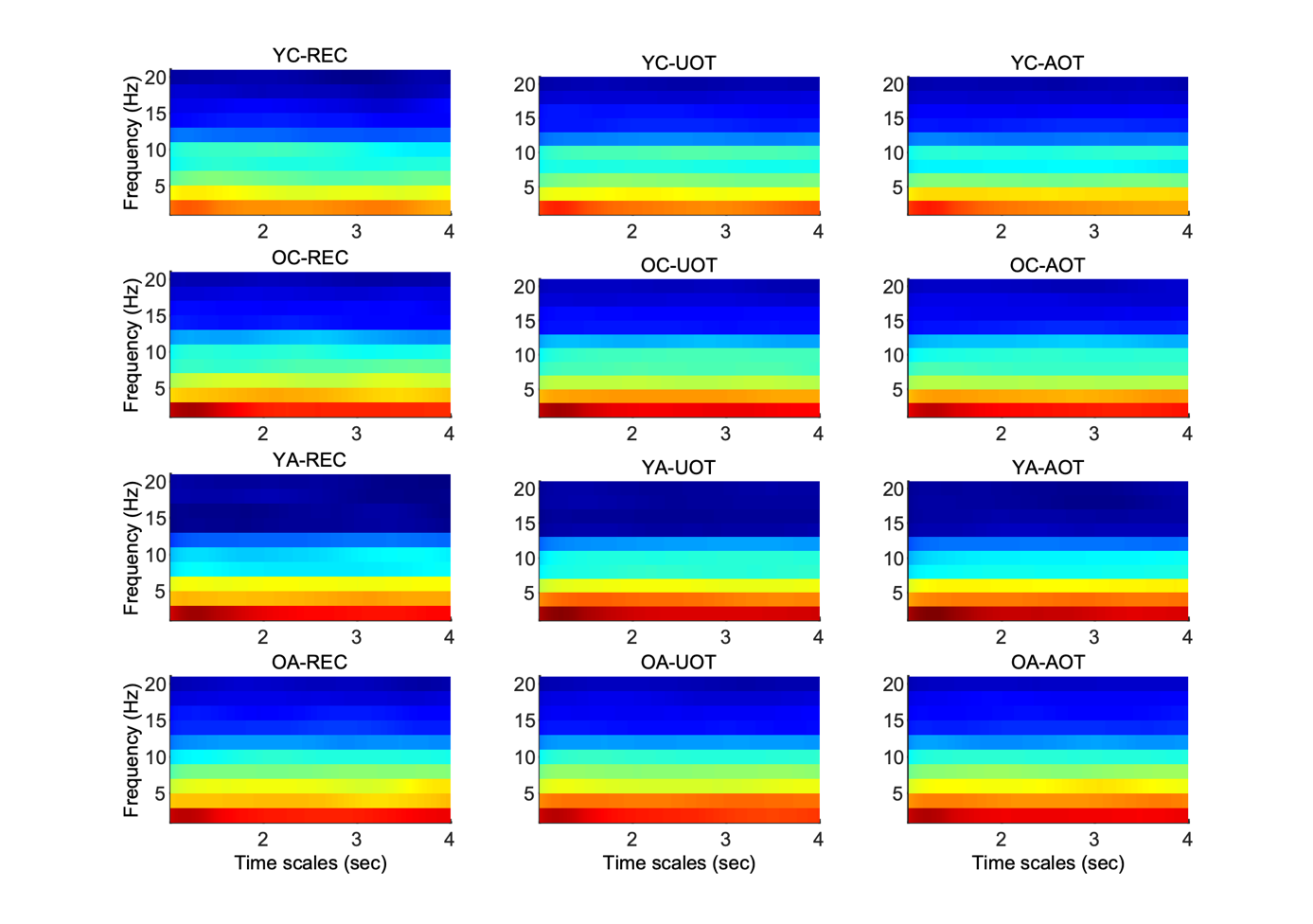


**S4 Fig. Group and condition means of *σJL*(τ,*f*).** Figure arrangement and conventions similar to **S2 Fig**

Supplement: S4 Fig — Figure arrangement and conventions similar to S2 Fig. (DOCX) [file pcbi.1013290.s004.docx]

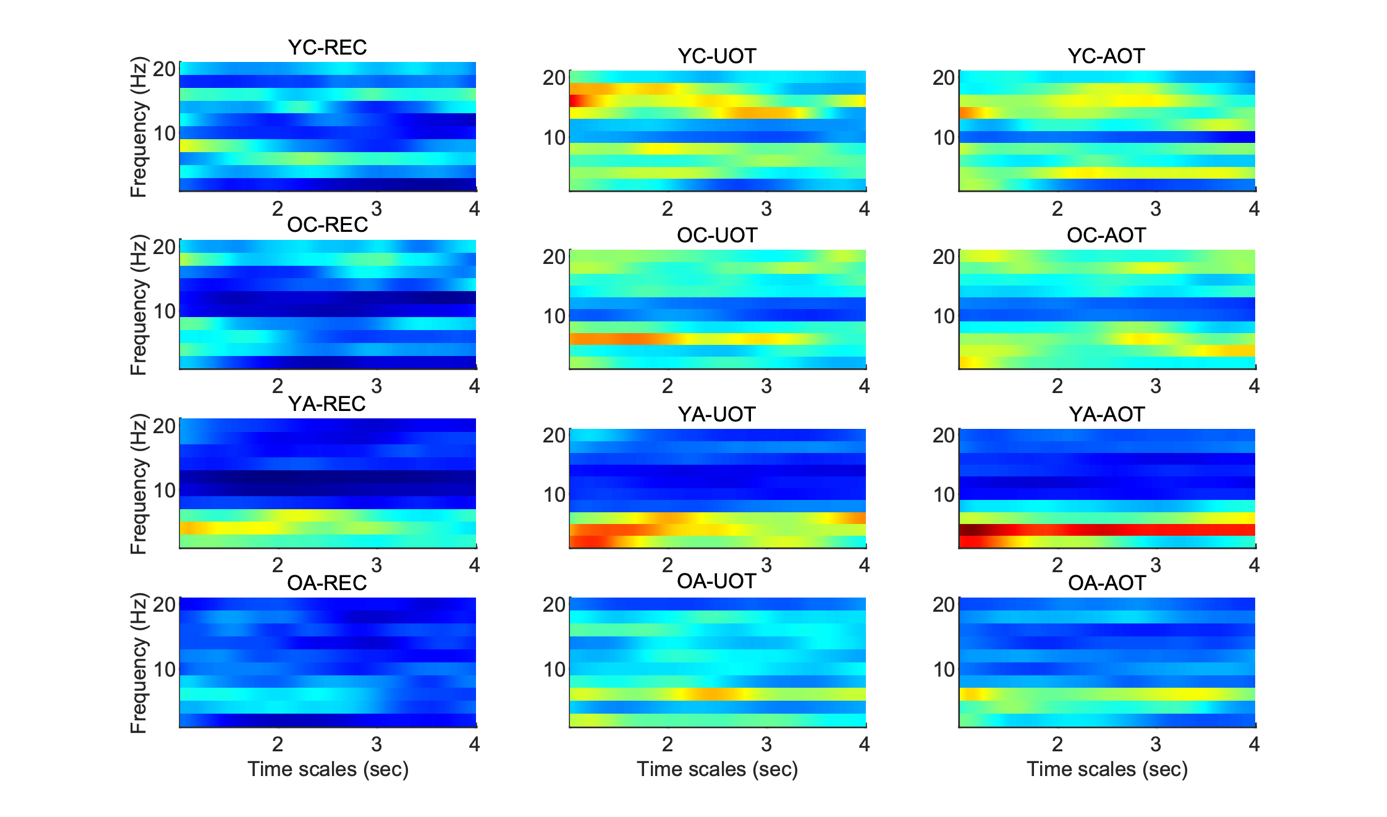


**S5 Fig. Group and condition t-score of *σJL*(τ,*f*).** Figure arrangement and conventions similar to **S2 Fig**.

Supplement: S5 Fig — Figure arrangement and conventions similar to S2 Fig. (DOCX) [file pcbi.1013290.s005.docx]

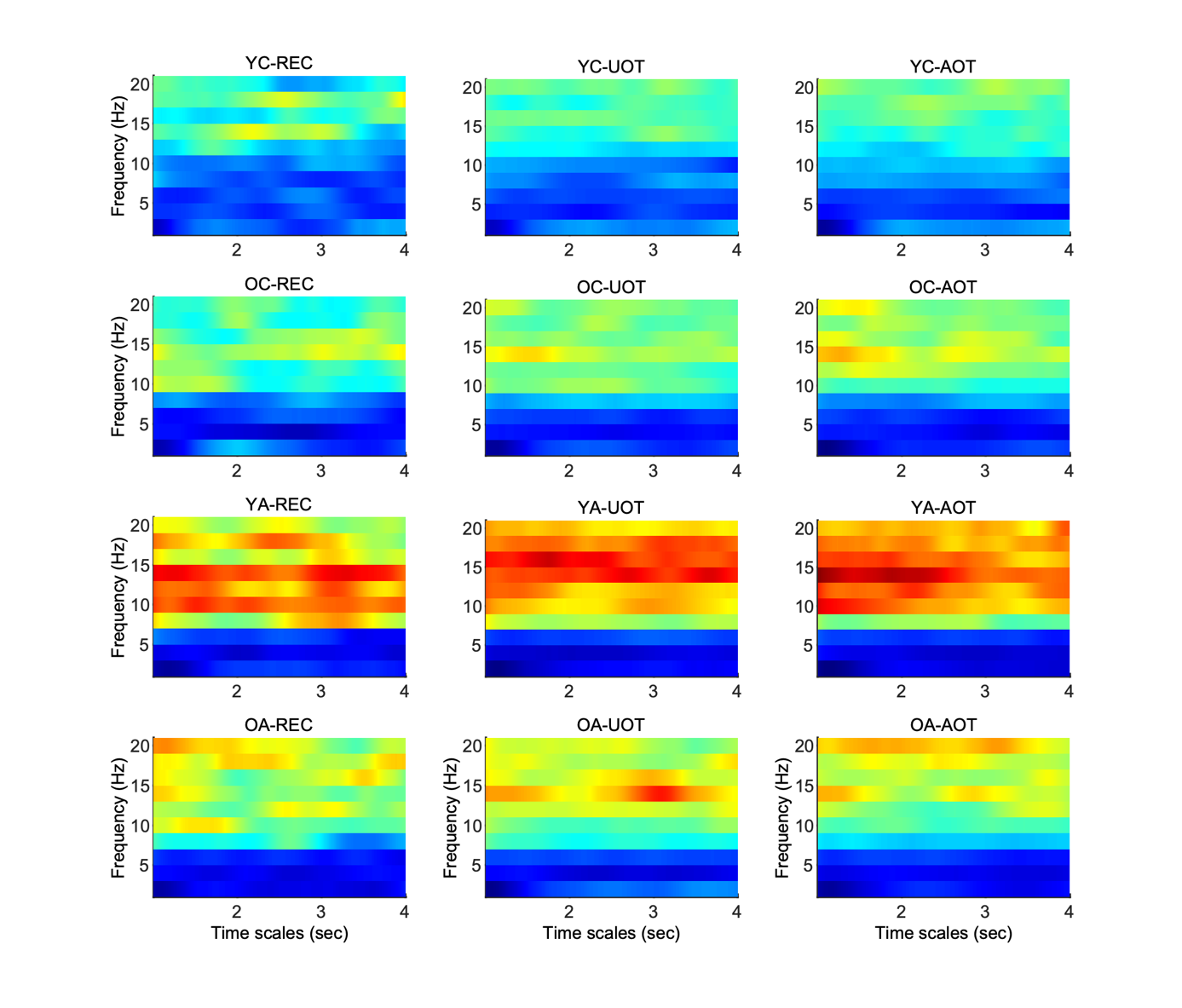


**S6 Fig. Group and condition means of *kJL*(τ,*f*).** Figure arrangement and conventions similar to **S2 Fig**.

Supplement: S6 Fig — Figure arrangement and conventions similar to S2 Fig. (DOCX) [file pcbi.1013290.s006.docx]

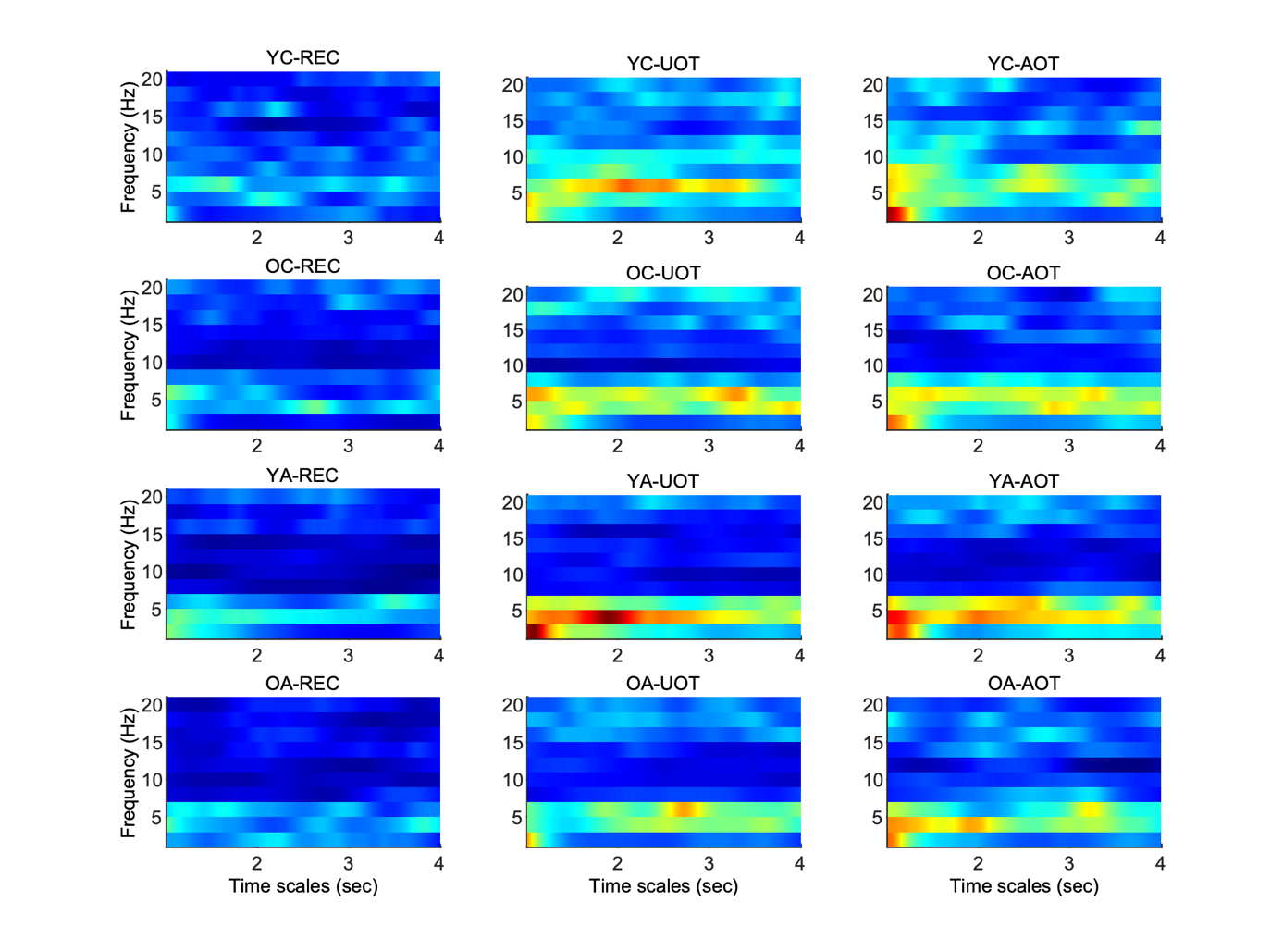


**S7 Fig. Group and condition t-score of *kJL*(τ,*f*).** Figure arrangement and conventions similar to **S2 Fig**.

Supplement: S7 Fig — Figure arrangement and conventions similar to S2 Fig. (DOCX) [file pcbi.1013290.s007.docx]
