# Supplementary material for: Developmental and aging changes in brain network switching dynamics revealed by EEG phase synchronization
Source: PLoS Comput Biol. 2026 Apr 16;22(4):e1013290. doi: 10.1371/journal.pcbi.1013290 (PMC13124065; doi:10.1371/journal.pcbi.1013290)
Supplement: S2 Fig — Groups are arranged in rows (age increases from top to bottom) and conditions in columns (attentional demands increase from left to right, i.e., ‘REC’, ‘UOT’, ‘AOT’). Time scale in seconds for all x-axes and frequency in Hz for y-axes. Note the transient effect for approximately the first second that corresponds to the length of the –fully overlapping- sliding window of phase synchronization computation. Only data for timescales in the range 1.004-2 sec, almost 1 sec after the length of the overlapping window, were used for further statistical analysis. (DOCX) [file pcbi.1013290.s002.docx]

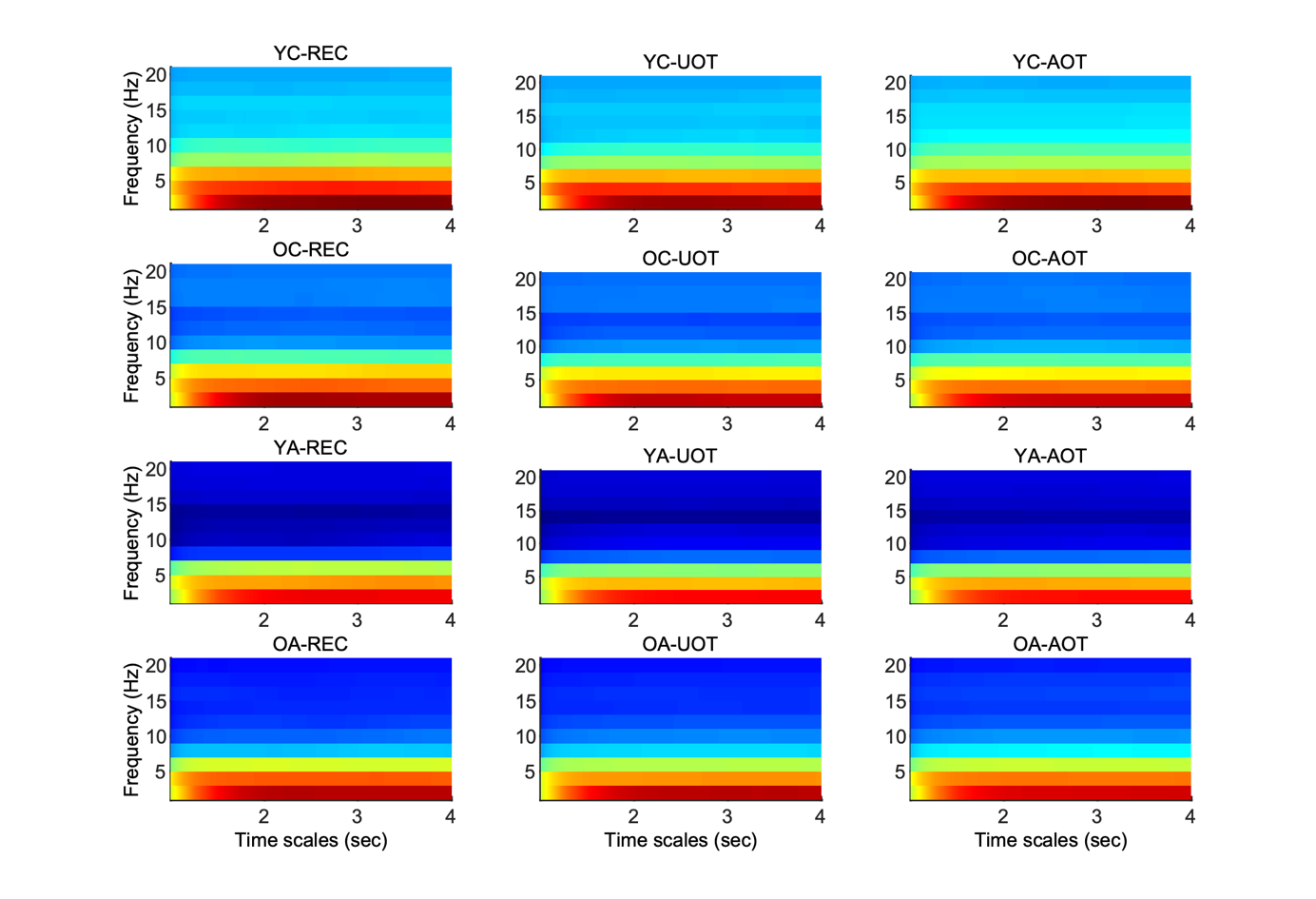


**S2 Fig. Group and condition means of *μ_JL_*_(τ,_*_f_*_)_.** Groups are arranged in rows (age increases from top to bottom) and conditions in columns (attentional demands increase from left to right, i.e., ‘*REC*’, ‘*UOT*’, ‘*AOT*’). Time scale in seconds for all x-axes and frequency in Hz for y-axes. Note the transient effect for approximately the first second that corresponds to the length of the –fully overlapping- sliding window of phase synchronization computation. Only data for time scales in the range 1.004-2 sec, almost 1 sec after the length of the overlapping window, were used for further statistical analysis.
