# Supplementary material for: Developmental and aging changes in brain network switching dynamics revealed by EEG phase synchronization
Source: PLoS Comput Biol. 2026 Apr 16;22(4):e1013290. doi: 10.1371/journal.pcbi.1013290 (PMC13124065; doi:10.1371/journal.pcbi.1013290)
Supplement: S8 Fig — Top row: Each panel shows the weights of the second task latent (asterisks), and the normalized brain scores with 95% confidence intervals (bars and errorbars) variable σJL(τ,f) (left) and kJL(τ,f) (right). Color and other conventions are identical to Fig 10 of the main text. This LV is significant (p < 0.003 for σJL(τ,f) and p < 0.006 for kJL(τ,f)) and explained approximately 33.3% and 6.8% of the covariance, respectively It contrasts ‘OC’ and ‘OA’ to ‘YC’ and ‘YA’ –in general- with statistical reliability (non-overlapping confidence intervals) for σJL(τ,f). Instead, the contrast described by the respective LV for kJL(τ,f) is not reliable at all. Bottom row: Each panel shows the bootstrap ratios of the brain latent variable of σJL(τ,f) (left) and kJL(τ,f) (right). Positive (negative) values correspond to elements that correlate positively (negatively) with contrasts of the top row, i.e., where that young adults (children) have higher (lower) values, respectively. Only values at 8 Hz for σJL(τ,f), and at 16 and 20 Hz for a short range of scales around 1.6 sec and 1.85 sec respectively, for kJL(τ,f), have bootstrap ratios with absolute values greater than 2.5758. Therefore, these contrasts are statistically unreliable in general, and are not discussed in the main text. (DOCX) [file pcbi.1013290.s008.docx]

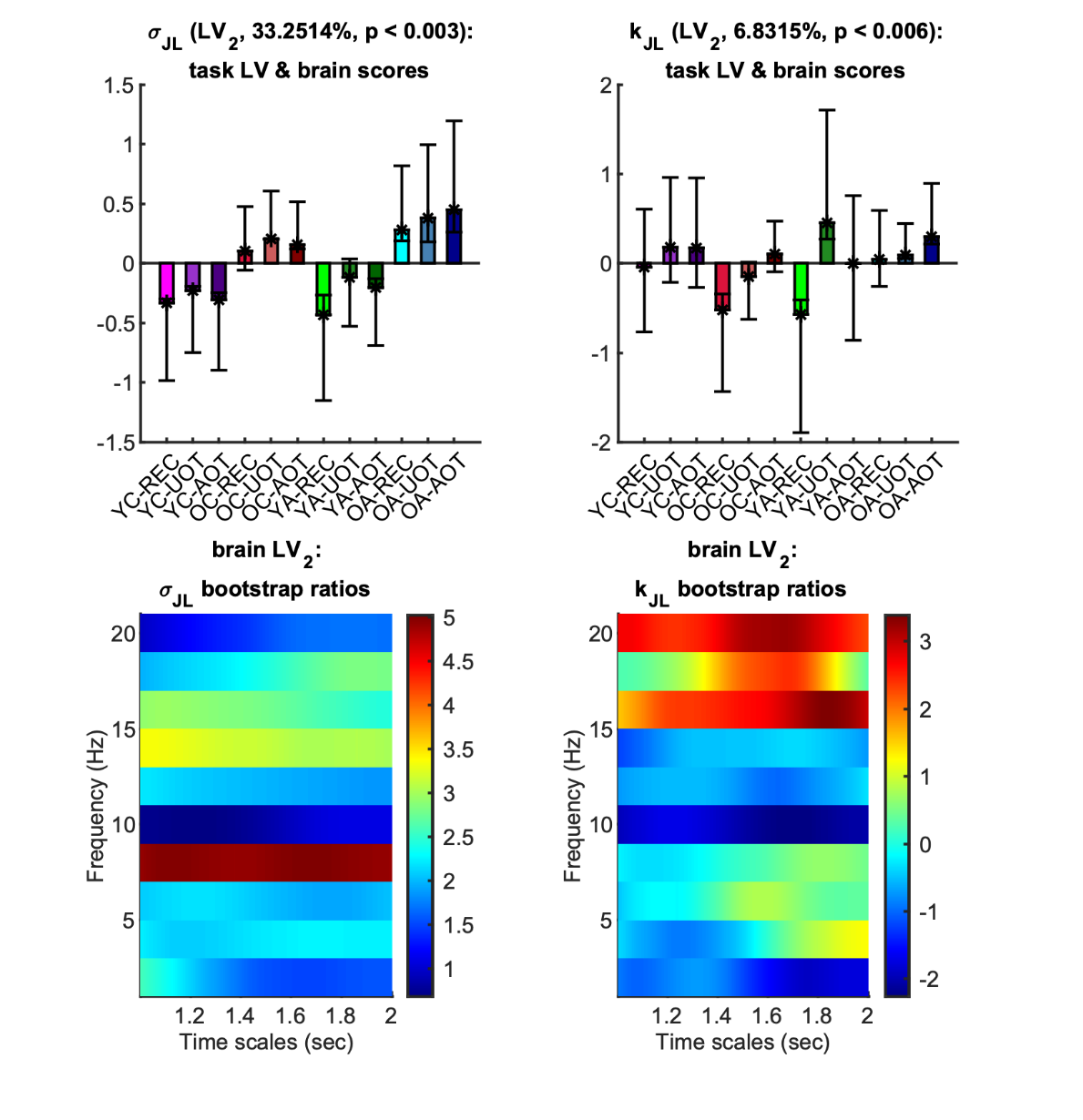


**S8 Fig. Second task latent variable, normalized brain scores and brain latent variable of the phase synchronization dynamics metrics.** *Top row:* Each panel shows the weights of the second *task latent* (asterisks), and the normalized brain scores with 95% confidence intervals (bars and errorbars) *variable* *σ_JL_*_(τ,_*_f_*_)_ (left) and *k_JL_*_(τ,_*_f_*_)_ (right). Color and other conventions are identical to *Fig 9* of the main text. This LV is significant (*p* < 0.003 for *σ_JL_*_(_*_τ_*_,_*_f_*_)_ and *p* < 0.006 for *k_JL_*_(_*_τ_*_,_*_f_*_)_) and explained approximately 33.3% and 6.8% of the covariance, respectively It contrasts ‘*OC*’ and ‘*OA*’ to ‘*YC*’ and ‘*YA*’ –in general- with statistical reliability (non-overlapping confidence intervals) for *σ_JL_*_(τ,_*_f_*_)_. Instead, the contrast described by the respective LV for *k_JL_*_(τ,_*_f_*_)_ is not reliable at all. *Bottom row:* Each panel shows the bootstrap ratios of the brain latent variable of *σ_JL_*_(τ,_*_f_*_)_ (left) and *k_JL_*_(τ,_*_f_*_)_ (right). Positive (negative) values correspond to elements that correlate positively (negatively) with contrasts of the top row, i.e., where that young adults (children) have higher (lower) values, respectively. Only values at 8 Hz for *σ_JL_*_(τ,_*_f_*_)_, and at 16 and 20 Hz for a short range of scales around 1.6 sec and 1.85 sec respectively, for *k_JL_*_(τ,_*_f_*_)_, have bootstrap ratios with absolute values greater than 2.5758. Therefore, these contrasts are statistically unreliable in general, and are not discussed in the main text.
