# Supplementary material for: Developmental and aging changes in brain network switching dynamics revealed by EEG phase synchronization
Source: PLoS Comput Biol. 2026 Apr 16;22(4):e1013290. doi: 10.1371/journal.pcbi.1013290 (PMC13124065; doi:10.1371/journal.pcbi.1013290)
Supplement: S1 Table — (DOCX) [file pcbi.1013290.s009.docx]

**S1 Table. The 19 EEG electrodes used for the phase synchronization computation in the original 10-10 nomenclature and their mapping to 10-20 system equivalents.**

| **Label 10-10** | **10-20 Equivalent** |
| --- | --- |
| Fp1, Fp2 | Fp1, Fp2 |
| Fz, Cz, Pz | Fz, Cz, Pz |
| F3, F4, F7, F8 | F3, F4, F7, F8 |
| C3, C4 | C3, C4 |
| T7, T8 | T3, T4 |
| P7, P8 | T5, T6 |
| P3, P4 | P3, P4 |
| O1, O2 | O1, O2 |

See the International Federation of Clinical Neurophysiology (Nuwer et al., Electroencephalogr Clin Neurophysiol., 1998).
