# Supplementary material for: Developmental and aging changes in brain network switching dynamics revealed by EEG phase synchronization
Source: PLoS Comput Biol. 2026 Apr 16;22(4):e1013290. doi: 10.1371/journal.pcbi.1013290 (PMC13124065; doi:10.1371/journal.pcbi.1013290)
Supplement: Attachment — Submitted filename: ResponseToTheReviewers.docx [file pcbi.1013290.s011.docx]

Dear Editors and Reviewers,

Thank you for the opportunity to resubmit a revised version of our manuscript for publication in PLoS Computational Biology. We sincerely appreciate the time and effort that both the editors and reviewers have invested in reading our manuscript and providing valuable feedback.

We have carefully considered the reviewers' suggestions and have updated our manuscript accordingly. We believe that these revisions have significantly improved the quality of our work. Moreover, additional effort was made to improve the overall readability and conciseness of the text. To facilitate the review process, we have provided: (i) a revised version of the manuscript and (ii) a manuscript version with tracked-changes, and (iii) this point-by-point response to each comment below (reviewers’ comments in blue font and our answers in black font). Each significant change in the main text resulting from reviewers’ comments is explicitly mentioned in our responses in this document with reference to the modifications in the revised manuscript with tracked changes.

We are grateful for the opportunity to improve our work based on the suggestions provided by the reviewers. We look forward to your further consideration of our revised manuscript.

Best regards,

Dr. Dionysios Perdikis

**Responses to reviewers’ comments**

- **Reviewer #1:**

“I think this is a high-quality and impactful work. The results offer new insights into brain aging, and help bridge the gap between neuroimaging and the mechanistic research at the micro- and mesoscales. The authors achieve this through robust time-series analyses of EEG data from a sample with an exceptionally wide lifespan coverage (9–75 years). Using a set of metrics based on activity and phase synchronization, they identify distinct age-related patterns of monotonic trends along with features that show inverted U-shaped trends, helping to dissociate brain development and aging effects. The paper overall is well written and the concepts are clearly articulated. I particularly appreciated the effort to include simplified explanations alongside complex concepts, these helped a lot with readability. My comments are only minor and primarily focus on further improving the clarity of the work. I believe this paper would be an impactful contribution and a good fit for PLOS Computational Biology.”

1. “I understand there were three different scans (rec, uot, aot) from each participant, and that they were considered simultaneously in the PLS analyses. Could you provide some assurance that the task-related effects didn’t confound the age-related ones? Would the identified age-related trends persist if multiple instances of PLS were run separately for the different tasks? (no need to carry out these analyses if the answer is simple)”

*First, we wish to thank the reviewer for the overall positive and constructive feedback.*

*We understand the concerns of the reviewer. It should be noted however that mean centered PLS does not perform any test for a specific contrast, neither a task nor a group related one. The contrasts tested via the permutation test for statistical significance correspond to specific latent variables (LVs), as shown in the respective figures (i.e., Fig. 3, 5 and top row panels of Fig. 8 and 9). They are derived by means of singular value decomposition. As depicted in the figures, all those LVs obviously separate age groups with minor differences for tasks, which are not statistically reliable (since the confidence intervals of the bars overlap). Therefore, the way the analysis is run, we don’t think that there is any possibility of confounding group effects with task effects.*

2. “Relatedly, did you notice any trends with respect to the task? Do the uot/aot tasks represent a heavier cognitive load? If so, does this show up in any of the metrics you looked at?”

*Mean centered PLS did not return any statistically significant latent variable contrasting tasks. Moreover, since task differences were not central to our research question, we refrained from discussing it any further in the absence of statistically significant task trends in our metrics.*

3. “I think the presented simulations were valuable in proposing mechanistic hypotheses, and I felt these results could be discussed in a bit more detail to better motivate follow-up experimental work. Particularly, a short discussion of how the model parameters correspond to specific mechanisms would be useful.”

*The model should be understood as capturing how lifespan-related physiological changes collectively shift the brain between regimes of weak, intermediate, and strong effective coupling, rather than attributing observed dynamics to any single mechanistic parameter. We can thus not identify a single mechanism but can interpret the dynamic regimes. Within this framework, the intermediate regime that reproduces the empirically observed switching dynamics corresponds to a metastable balance between regional autonomy and network-level coordination. To aid in this understanding, we added the following text in the revised manuscript (lines 708-723):*

“The oscillator model used here is intentionally phenomenological and is not designed to map each parameter onto a single specific biological process. Instead, the parameters represent effective dynamical control variables governing large-scale synchronization. The connectivity matrix constitutes the only explicitly anatomical component of the model, as it is derived from diffusion-MRI tractography and therefore reflects the structural white-matter scaffold constraining inter-regional communication. By contrast, the global coupling parameter controls the overall strength of interactions across this scaffold and should be interpreted as an aggregate measure of effective inter-regional communication efficacy. Such efficacy may arise from multiple biological sources, including synaptic gain, neuromodulatory tone, excitation–inhibition balance, or conduction reliability, rather than a single identifiable mechanism. Similarly, the local stability parameter regulates how strongly each oscillator tends to remain in or depart from a phase-locked configuration; at a biological level this corresponds broadly to regional excitability and the tendency of neuronal populations to engage in synchronized activity, again reflecting a compound effect of multiple microscopic processes. A characterization of neurodegeneracy and mapping specific biological parameters on reduced dynamical parameters is an objective of virtual brain twin approaches [86-88] and paves the road to applications in personalized medicine.”

4. “Do you have any thoughts on how the inverted U-shaped trends in power/MSEn may relate to the inverted U-shaped trend in phase synchronization dynamics? If I missed this in the text, I’d appreciate a pointer to it.”

*The correspondence between power (P)/MSEn and PSD inverted U-shaped trends was indeed not explicitly discussed in the manuscript to avoid speculations. However, since the reviewer asked for an interpretation, we have now added a paragraph to the Conclusion section addressing the possible shared underlying mechanism (lines 737-750).*

“An inverted U-shaped trend component was also observed for power/MSEn changes. This likely reflects a similar underlying lifespan reconfiguration of large-scale networks—maximal distributed, flexible coordination in young adulthood—seen from a “local signal” perspective (power/MSEn) and a “global network dynamics” perspective (phase-synchrony state switching). On the one hand, the changes in P and MSEn in the 6–24 Hz range can be interpreted as reflecting a lifespan peak in the local repertoire and temporal differentiation of oscillatory activity, particularly in posterior and fronto-central regions (McIntosh et al., 2013). On the other hand, and in comparable frequency bands, PSD changes suggest a lifespan peak in the exploration of functional network states, with young adults showing controlled, yet flexible, exploration regime. The convergence of these patterns suggests that maximal local complexity (indexed by power/MSEn) around mid-frequencies could be a prerequisite for a maximal flexibility and efficiency of larger-scale synchronization dynamics, both of which peak in young adulthood and decline at the two ends of the lifespan (Douaud et al., 2014). In other words, the optimal level of local variability in young adults constitutes an oscillatory drive that enables the brain to sample many phase-synchronization configurations without either undersampling (rigidity) or oversampling (instability).”

5. “I think the line between what literature already exists and what this work is adding could be more explicitly defined in the introduction.”

*We thank the reviewer for pointing out this shortcoming in the Introduction. We edited the text of the last two paragraphs of the Introduction to make the gap in the literature more explicit. We specifically added the following sentences:*

*Lines 151-155*: “However, these two different approaches have typically been pursued in isolation, such that stationary and dynamic measures, and linear and non-linear metrics, have not been systematically integrated, especially not within the same lifespan study. As a result, we still lack a unified empirical account of how monotonic and non-monotonic changes in neural variability relate to each other and to underlying mechanisms across development and aging."

*Lines 176-179*: “The adopted integrative data analysis approach together with the modelling strategy extends prior work by linking static and dynamic EEG markers, linear and non-linear metrics, as well as empirical and generative levels of description in a unified account of development and aging effects on brain signal variability and dynamics."

6. “For the phase synchronization analyses, on what criteria were the 19 regions selected, and where can I find a list of these regions?”

*We apologize for not specifying this in the initial manuscript. For the phase synchronization analyses, we used only 19 electrode locations from the 10–20 system of the International Federation of Clinical Neurophysiology (Nuwer et al., Electroencephalogr Clin Neurophysiol., 1998) to avoid volume conduction effects between electrode sites located close together. We have also added a table (Table S1) in the revised Supplementary Information with the 19 electrodes’ labels in the 10-10 system and their mapping to the 10-20 system. All this is now specified in the Methods section of the revised manuscript (lines 277-279):*

“For the phase synchronization analyses, we selected N_ch_ = 19 channels out of the available 58, based on the 10–20 system (see Table S1 in Supplementary Information), to avoid volume conduction effects between electrode sites located close together.”

7. “The PLS step and its justification make sense, but it took me several reads to understand. I’d suggest considering a figure with a schematic explaining this step. I leave this to your judgment. It could potentially be added as a panel in Figure 1, if there’s space.”

*We understand the difficulty to fully grasp PLS. However, since there are publications explaining the PLS method in quite detail and we cite them, we would prefer not to overload our paper with the proposed figure. Moreover, Figure 1 is already very clattered.*

8. “Figure 1 appears low resolution on my end, even when accessing the raw TIF file. If this is inherent to the figure, I’d suggest regenerating it in higher resolution, some details are hard to read.”

*We have regenerated Figure 1 with the PLoS Comp. Biol. specification of 300 dpi resolution.*

9. “Despite the caption referencing it, I couldn’t locate the “thick dotted black line” in Figure 1, panel F.”

*We have added the dotted line back using red color for better visibility, and we accordingly adjusted the legend of Figure 1.*

10. “On Figures 2 and 7, it would be helpful to include x-tick labels in every row. Having to constantly look across the figure makes it harder to navigate.”

*We have added all missing x-tick labels in Figures 2 and 7.*

11. “The percentages shown in Figures 2–6 and 8–9 seem overly precise in my opinion and are a bit distracting”

*We have rounded all percentages in Figures to one decimal point just like in the main text.*

12. “I couldn’t find a definition for the acronym “LV”; I assumed it stands for latent variable, but it would be good to add this explicitly.”

*We thank the reviewer for noticing this problem. We added the term explicitly (i.e., latent variable”) upon its first appearance in the Methods section, at the paragraph on PLS (line 349). In the Results section, the acronym was already defined at its first appearance (line 432).*

**Reviewer #2:**

“This manuscript examines age trajectories in several EEG features, as indicators of changes in state dynamics. A key finding is that both monotonic and inverted-U (non-monotonic) trajectories were found, coexisting within the feature variance, and consistent with multiple system dynamics changing independently across age. The manuscript is overall clearly presented, methods are rigorous, and the perspective is innovative, offering a multivariate perspective on brain state dynamics across age, and including a simulation model based on oscillator coupling, that could have broad interest. Some concerns in the current versions are outlined below, and include consideration of confounds, as well as sparsity in some analytical details and rationale that could affect the impact or interpretation of results.”

Considerations:

1. “The sample size of n=111 is not large for population scale inferences about development, especially because it is cross-sectional not longitudinal. This is a known limitation of population studies, and will of course affect the statistics (e.g., the generalizability of the cross-covariance PLS matrices, based on n=24-31 individuals per group). This ought to be discussed in the caveats.”

*We are overall grateful for the reviewer’s comments that helped us improve the clarity of our manuscript and expand its outreach.*

*Regarding sample size, it can indeed be considered a limitation in our study, even though we attempted to reduce its incidence on the statistical results through using permutation and bootstrap tests. We acknowledge this limitation in the revised Conclusion section (lines 754-761):*

“We point out that sample size can be considered a limitation of the present cross-sectional study, particularly for population-level inferences about lifespan development and aging, even though we sought to mitigate its impact on the statistical results by relying on permutation and bootstrap procedures; this caveat should be borne in mind when extrapolating our findings beyond the sampled cohorts. A critical next step is to extend this approach to larger samples while including simultaneous behavioral recordings, allowing us to trace how brain and behavior co-evolve across the human lifespan.”

2. “The pre-processing for EEG is omitted, with references provided instead. Given that cleaning protocols may impact the features that are at the heart of the analyses presented, these protocols ought to be presented in full. This is particularly important because whether and how much there are confounding signals contributing to the results, cannot be evaluated without such information (or perhaps relate to why the EO condition is different and omitted).”

*We thank the reviewer for pointing this out. We added the preprocessing steps in the revised manuscript as suggested (lines 214-218), together with the important precision that task conditions were performed with eyes closed (lines 196-198).*

*Lines 214-218:* “The left mastoid was used as a reference, and the right mastoid was recorded as an active channel. The data were re-referenced off-line to an average of the left and right mastoids for further analysis. Vertical and horizontal electrooculograms were recorded for control of eye blinks and eye movements. Eye movement correction was accomplished by independent component analysis [67]. Thereafter, artifacts from head and body movements were rejected by visual inspection.”

*Lines 196-198:* “The EEG measurement began with a 3-minute resting state recording (1.5 minutes with eyes closed, and 1.5 minutes with eyes open) and was followed by the auditory oddball task performed with eyes closed.”

3. “It is somewhat curious that EO was omitted due to a “different power profile”. This is notable because this condition is more akin to task than the EC condition. Given that the groups effects are common across tasks, this suggests the possibility that some odd confounding property of the signal could be contributing to the similarity between EC and task. An example is amount of blinks in the data or noise. Perhaps the task differences could be more fully justified and explained in the supplemental materials.”

*We understand the reviewer’s concern, which is mainly caused by the ambiguity we inadvertently left in the Methods section. As mentioned above, we now clarify in the revised manuscript that all task conditions were performed with eyes closed (EC). Therefore, the EO (eyes open) condition is not actually more comparable to the task conditions than the EC condition. In fact, the protocol allowed us to control that both the resting EC condition and all task conditions share the same visual state, which reduces the likelihood that systematic differences in ocular activity (e.g., blinking) or related artifacts could account for the observed effects.*

*Furthermore, all datasets underwent identical preprocessing procedures, including artifact correction and rejection steps, which should minimize residual confounding effects related to signal quality or noise. Given this, we consider it unlikely that the similarity between EC and task conditions reflects an odd confounding property of the signal. Moreover, according to the permutation tests, there was no statistically significant latent variable (LV) of the mean centered PLS with statistically reliable (based on the bootstrap test) differences among the three tasks investigated.*

4. “Relatedly, it would be helpful to see assessments of the data quality, or the results of data cleaning. It is always a concerns that the age effects are because children move more or blink less, affecting the entire time series via confound signals not neural state changes.”

*The reviewer’s concern regarding potential age-related differences in data quality (e.g., movement or blinking artifacts) that might confound group effects is legitimate. To mitigate this, in the revised Methods section, we now provide a more explicit description of the preprocessing pipeline (lines 214-218). Vertical and horizontal electrooculograms (EOG) were recorded to capture eye blinks and eye movements, and ocular artifacts were corrected using independent component analysis (ICA). In addition, artifacts related to head and body movements were identified and removed through careful visual inspection. These procedures were applied consistently across all age groups.*

*Given these preprocessing steps, we consider it unlikely that the observed group effects can be attributed to systematic differences in data quality rather than neural dynamics. While we acknowledge the general importance of reporting data quality metrics, the preprocessing procedures implemented here follow established standards in EEG research and were designed specifically to minimize non-neural confounds.*

*For these reasons, we believe that additional retrospective reconstruction of preprocessing outputs is unlikely to substantially alter the interpretation of the findings. Nonetheless, we have clarified the preprocessing description in the manuscript to make these safeguards more transparent.*

5. “Finally, also related to 2-4 above, it would be valuable to know how these different features relate to one another? Would variance and MSE be considered redundant through mathematical relationships? How about phase coupling, or relationships between PSD and MSE? Note that across features the first LV shows a monotonic change and the second LV shows a non-monotonic change - would this be a side effect of these measures all being related to one another?”

*It is indeed the case that spectral power P relates to variance at different time scales, and therefore there is some redundancy between P and standard deviation σ. Moreover, in a previous study, we have shown how MSE and P (or other measures of linear autocorrelations) relate to each other (Courtiol et al 2016; cited in the Manuscript). Although MSE tracks changes to linear autocorrelations across scales as well, it is also more sensitive to nonlinearities. Nevertheless, we cannot form an informed hypothesis about the structural relationship of the local, univariate measures and the network metrics. Such a hypothesis would have to rely on another hypothesis about the exact nature of the brain network dynamics itself, for which our current results are not adequate. It is true that the relationship between P and MSEn could indeed explain why they both show a first monotonic LV and a second U-shaped one. However, we cannot see a structural relationship with the first monotonic LV of the phase synchronization measure. Finally, the PSD measures show only the U-shaped LV. In summary, despite some overlap, we don’t think that there is a full redundancy among the measures, except for between P and σ. We have added a short comment acknowledging this in the Discussion (lines 643-647):*

“Finally, we should note that the common trends among all four univariate measures for LV1, as well as for the LV2 for P and MSEn, could reflect linear autocorrelation properties of the EEG signals, rendering them partially redundant. This is true mainly for P and σ, since MSE and MSEn have been shown to be more sensitive to nonlinear properties of signals [41, 43, 71].”

6. “The explanations of core features (e.g., MSE, extended MSE) and concepts (stationarity & why PSD is non-stationary) in the introduction could be expanded to make the rationale and predictions more explicit. The significance of the simulation and choice of coupled oscillations is similarly not entirely clear. Why is this particular model valuable?”

*We thank the reviewer for highlighting the need to better motivate the different concepts and metrics used, as well as clarifying the role and the choice of the coupled oscillators’ simulations.*

*Regarding the metrics, we have now made modifications and additions to the last paragraph of the Introduction to better explain how the concepts of nonlinearity and non-stationarity of the dynamics of brain measures relate to our methodological choices (lines 162-173):*

“Stationary features, i.e., those assumed to be stable across the time of the EEG measures, are quantified with linear (spectral power and standard deviation) and nonlinear (multiscale entropy) metrics and multivariate statistics, whereas non-stationary features are captured through time-resolved phase-synchronization dynamics, using multivariate statistics in all cases. The use of such a battery of measures is motivated by a hypothesis, associated with an inverted U-shape trajectory across age groups, that the healthy, mature, adult brain balances optimally the stability of a variety of dynamical modes and the flexibility to switch among them. Such dynamics is more likely to be evaluated by dynamic metrics, which focus on the transitions among distinct modes, than by static ones that rely on averaging across time, since no brain measurement is long enough to allow for any time averaged statistic to capture the structure of the total state space. Accordingly, nonlinear metrics are expected to be more sensitive to such non-stationary dynamics, since transitions between functional modes is a definite signature of nonlinear dynamics.”

*Moreover, we stressed further the nonlinear character of MSE, and, especially, MSEn, via a small addition to the end of the following sentence in Methods (lines 241-243):*

“A previous study [41] has shown that this modified metric provides additional information on EEG variability (see also [71]) for a similar approach), focusing more on nonlinear properties of the underlying dynamics than MSE.”

*Similarly, we added the following lines (328-330) in the Methods for the description of the PSD metrics:*

“The PSD metrics allowed us to statistically evaluate the dynamics of transitions in time among distinct phase synchronization networks, which roughly correspond to different modes of brain dynamics, as opposed to all other metrics used in this study that rely on averaging across time.”

*As far as the model employed here is concerned, it is intentionally phenomenological but incorporates an essential mechanistic ingredient using empirically derived structural connectivity, which constrains the topology of interactions, and thereby shapes the collective phase dynamics of the network. In this sense, the simulations do not aim to reproduce detailed EEG signals or to map parameters onto specific biological processes, but rather to probe the dynamical regimes that emerge when anatomically constrained oscillatory populations interact. The principal value of the model is therefore interpretational rather than predictive: it establishes a dynamical bridge between abstract coupling regimes and the observable signatures of synchronization variability measured empirically. In doing so, it clarifies that the lifespan-dependent differences observed in the data can be understood as shifts of the large-scale brain system along a continuum from under-coordinated dynamics, through a metastable regime supporting a rich functional repertoire, toward overly stabilized (rigid) coordination with reduced dynamical flexibility.*

*To further clarify in the manuscript, we added the following in our revision (lines 697-723)*

“From a dynamical-systems perspective, our oscillator model provides a controlled reference for interpreting the synchronization metrics used in the empirical analysis. It is expected that very weak coupling yields largely independent oscillators with little synchronization, whereas very strong coupling leads to rigid phase locking. However, central to the present study, the model illustrates how these different coordination regimes express themselves in the statistical signatures of network switching quantified in the data. The simulations demonstrate that only an intermediate coupling regime produces a metastable landscape in which multiple partially synchronized network configurations coexist and are intermittently explored. This regime generates slow, structured transitions among network states and a broad distribution of switching events, precisely the type of behavior captured by the jump-length statistics and topology-based synchronization dynamics measures applied to the EEG recordings.

The oscillator model used here is intentionally phenomenological and not designed to map each parameter onto a single specific biological process. Instead, the parameters represent effective dynamical control variables governing large-scale synchronization. The connectivity matrix constitutes the only explicitly anatomical component of the model, as it is derived from diffusion-MRI tractography and therefore reflects the structural white-matter scaffold constraining inter-regional communication. By contrast, the global coupling parameter controls the overall strength of interactions across this scaffold and should be interpreted as an aggregate measure of effective inter-regional communication efficacy. Such efficacy may arise from multiple biological sources, including synaptic gain, neuromodulatory tone, excitation–inhibition balance, or conduction reliability, rather than a single identifiable mechanism and illustrates the degeneracy present in multiscale systems. Similarly, the local stability parameter regulates how strongly each oscillator tends to remain in, or depart from, a phase-locked configuration. At a biological level, this corresponds broadly to regional excitability and the tendency of neuronal populations to engage in synchronized activity, again reflecting a compound effect of multiple microscopic processes. A characterization of neurodegeneracy and mapping specific biological parameters on reduced dynamical parameters is an objective of virtual brain twin approaches [86-88] and paves the road to applications in personalize medicine.”

7. Lines 666-668 state that there exists a differentiation between monotonic and non-monotonic patterns as synonymous with static and non-static measures but this is not quite in line with the findings, which do also find (in LV2) non-monotonic effects for “static” measures (MSE, P).

*We thank the reviewer for noticing this error. Indeed, that statement in the Conclusion did not summarize our results accurately enough. We now changed it to better reflect the fact that the “static” measures exhibit primarily a monotonic effect with group age, as illustrated by the LV1 explaining most of the variance. It is the other way around for the PSD metrics for which most of the variance is captured by the U-shape effect. We now state that (lines 728-731):*

“Aggregate, “static” indices exhibit primarily a roughly linear decline from childhood to older adulthood, explaining most of the variance, whereas transient, time-resolved metrics of network switching follow a pronounced inverted U-shaped trajectory, peaking in young adulthood.”

Minor notes:

- “The figures lack resolution - e.g., figure1 text is hard to read in places.”

*We have regenerated all figures with the PLoS Comb. Biol. specification of 300 dpi resolution.*

- “The functions used for extraction of features are inconsistently documented (listed in Fig 1 legend for some, but not all, and not in text).”

*Indeed, we mention explicitly the ksdensity() MATLAB function used to compute the probability distributions of jump lengths (JL(τ,f)) depicted in Figure 1 panel H and the cluster.kmedoids function of Python3 package pyclustering used to cluster the transient phase synchronization networks, depicted in Figure 10, panel C. In all other cases, computations for data metrics were performed either with standard MATLAB functions or with MATLAB code authored by one of the co-authors, Dr. Dionysios Perdikis, using standard MATLAB functions and following the equations described in the main text in detail. This code forms part of a toolbox (DPtoolbox), which includes also the PLS statistics’ code and has been made available in Zenodo at DOI: 10.5281/zenodo.15776182. Therefore, we think that further detailed references to MATLAB functions would not really add any clarity to the text. Finally, the only reference of those functions mentioned in figure legends, namely ksdensity() in Fig.1 legend (line 269), was removed, as there is indeed no need to repeat this detailed information in a figure legend.*

**Reviewer #3:**

“The authors present a fascinating study on lifespan trajectories of neural variability and synchronization using EEG and computational modeling. The integrated empirical-computational approach is a significant strength, and the findings regarding distinct monotonic and inverted U-shape trajectories are novel and of potential broad interest. The claim that metastable coupling underlies the optimal dynamics observed in young adults is compelling. However, several major issues need to be addressed before the manuscript can be considered for publication.”

Major Concerns:

1. The restriction of the EEG analysis to the 2-20 Hz range is a significant limitation that requires thorough justification. The authors must provide a clear, a priori rationale for excluding other relevant frequency bands. Why were the other high-frequency oscillations excluded? How do the authors ensure that the key lifespan trajectories they describe are not confounded by or do not manifest in these excluded frequencies? For instance, age-related changes in the power of other frequency bands are well-documented in the literature.

*We thank the reviewer for his enthusiasm and all the points he raised throughout his review.*

*Regarding the first raised point, our results on the univariate measures develop monotonously for higher frequencies (and faster time scales). This seems to be the case for all univariate measures (P, σ, MSE, MSEn), for both latent variables LV1 (Figure 4) and LV2 (Figure 6). Based on this observation, we restricted our analysis of phase sychronization (PS) and phase synchronization (PSD) dynamics to lower frequencies. However, indeed we could have extended the frequency range for those two measures to exclude the possibility of a trend reversal for higher frequencies. Considering retrospectively this point, we admit that this is a limitation of our study to be dealt with in future work, which we now mention in the Discussion (lines 678-680):*

“However, we should mention that future work should deal with a limitation in our present analysis, namely the restriction frequencies up to 20 Hz.”

2. “The manuscript states that analyses were performed on 19 electrodes, but it lacks a critical methodological detail: how and why these specific 19 electrodes were chosen from the original high-density (58-electrode) setup. Please specify the exact selection criteria (e.g., based on a standard 10-10 system subset, covering specific brain regions, or selected based on data quality).”

*We sincerely regret that we missed reporting this information in the initial submission. For the phase synchronization analyses, we used only 19 electrode locations from the 10–20 system of the International Federation of Clinical Neurophysiology (Nuwer et al., Electroencephalogr Clin Neurophysiol., 1998) to avoid volume conduction effects between electrode sites located close together. We have also added a table (Table S1) in the revised Supplementary Information with the 19 electrodes’ labels in the 10-10 system and their mapping to the 10-20 system. All this is now specified in the Methods section of the revised manuscript (lines 277-279):*

“For the phase synchronization analyses, we selected N_ch_ = 19 channels out of the available 58, based on the 10–20 system (see Table S1 in Supplementary Information), to avoid volume conduction effects between electrode sites located close together.”

3. “The computational model is a key component of the manuscript, but its current validation appears incomplete. The authors claim the model reproduced "the empirical combination of reduced low-frequency variability." However, the results seem to focus solely on replicating the phase-synchrony dynamics. The model's ability to replicate the univariate empirical findings (e.g., the monotonic decline in slow-frequency power, variance, and complexity) must be explicitly demonstrated. Does the model's output show the same age-dependent decline in these low-frequency measures?”

*The computational model proposed in this study is phenomenological and does not aim at replicating all empirical findings, but rather to guide interpretation of the metrics applied here. For instance, it cannot reproduce the frequency, multiscale content of the EEG signal. It only serves to illustrate the theoretical hypothesis we propose to interpret our empirical results based on brain dynamics “switching” between different modes of the brain’s dynamical repertoire for different time scales or frequency bands. It is mainly the statistics of this switching between different attractors of the phase synchronization dynamics against fluctutations in the neighborhood of each attractor that is captured in a qualitative manner only by the computational model. We removed the misleading claim that the model explains "the empirical combination of reduced low-frequency variability" from the abstract and we slightly modified that part of the text as follows (lines: 50-54):*

“Only an intermediate, metastable coupling regime reproduced qualitatively the empirical finding of maximally heterogeneous synchrony dynamics observed in young adults, whereas deviations toward weaker or stronger coupling mimicked the children’s and older adults’ profiles.”

*We think that the rest of the text, as well as the title of Figure 10 positions clearly our use of the model.*

4. “The model is used to explain differences between young adults, children, and older adults. However, the results lack a direct, visual comparison of the model's output for these specific groups against the empirical data. The authors should present a figure equivalent to Figure 9 (which nicely contrasts the empirical data across age groups) for the model's output. This would allow for a direct visual assessment of how well the model captures the distinct dynamical profiles of children and older adults compared to young adults.”

*As explained above, we use the model as a phenomenological illustration of how different patterns of PSD can arise, depending on an interplay between the strength of the nonlinear coupling and the stability of the network’s attractors, i.e., the model illustrates qualitatively a mechanistic explanation for the emergence of different patterns of PSD. Readers (as did we) will better appreciate our theoretical hypothesis for the potential explanation of our empirical findings. Therefore, the PSD matrices for different combinations of parameters a & K in Figure 10, panel B could be considered as – somewhat extreme for most of them- examples of distinct patterns of PSD. They are not aimed at being directly compared to PSDs’ patterns of different age groups and/or frequency bands, which is a level of comparison that would be more appropriate for Virtual Brain Twin level of modelling, which was not the aim of the current study. We now mention such developments at the end of the Discussion section before the Conclusion.*

Minor Concerns:

1. “In Figure 1, the x-axes in several panels lack units (e.g., likely "Hz" for frequency or "ms" for time). Please add the appropriate units to all axes for clarity.”

*We have added all x axes labels with units in Figure 1.*

2. “In Figure 10, Panels A and B are mentioned in the figure legend but are not referenced in the main text. The text should guide the reader through all panels of the figure.”

*We have modified the following part of the text of the Methods to now mention panels A and B of Figure 10 (lines 390-393):*

“**Fig. 10** depicts selected time series (*panel* A; from top to bottom $\cos\left( \theta_{i}\left( t \right) \right)$, $\cos\left( \theta_{i}\left( t \right)-\omega t \right)$ for the phase dynamics to be revealed and ${CCorr}_{i,j}\left( t,10 Hz \right)$) and $PSD$ matrices (*panel* B) for characteristic points of the above two dimensional parameters’ space.”
